# Report of Publication Committee

**Published:** 1894-11

**Authors:** L. H. Howard, A. T. Sellers

**Affiliations:** Philadelphia, Pa.; Philadelphia, Pa.


### Report of Publication Committee, United States Veterinary Medical Association.

Philadelphia, Pa., Sept., 1894.

Mr. President and Gentlemen: Your Committee would report that many difficulties were met with in completing the work assigned them, owing first to the lateness in which the matter for publication was placed in our hands. No fault, however, is attached to any officer of the Association, but due entirely to the stenographer, employed at Chicago; second, to the tardiness in which the proof sheets were returned to editor, thus delaying the publishing of the proceedings of the Chicago meeting nearly one year.

A motion was made and adopted at the last meeting, that one thousand copies of the proceedings be printed. Your Committee, after consulting the President and learning the condition of the treasury, deemed it advisable to print only six hundred and fifty copies, which were divided as follows: 200 hundred bound in cloth, 400 bound in paper, 50 copies remain in sheet for future binding as required. The total cost being $796.73, which includes printing, blnding, wrapping, postage and expressage. This means an expense on behalf of each member of $2, which entirely covers his annual dues, leaving nothing for the many other expenses. The initiation fee and annual dues must be raised, or we must curtail the work we are doing and cease publishing our proceedings.

Owing to the fact that we have annually, for the past three years, published the list of members in conjunction with the publishing of the proceedings, there has not been issued the annual list of officers and members, as provided for by the by-laws. A list of the officers and committees have been published as soon after the annual meeting as appointments were made and accepted. This has saved about $40 to $50 annually, and this should be allowed in conjunction with the cost of publishing the transactions. The cost of delivery this year has been lessened perhaps $50, by having, through the Smithsonian Library at Washington, D. C., all the copies delivered, postage and expressage free, and free from all tariff duties, under a special franking act, for the benefit of all scientific societies.

The question of the future employment of stenographers is one of much importance. While we have had enormous bills rendered for the service in the past, the work done has been unsatisfactory, incomplete, and ofttimes so slowly delivered to us that great delay has followed in the publishing of our proceedings as a result thereof.

We would recommend for the more prompt issue of your proceedings that no papers, reports, resolutions, etc., be accepted for consideration, unless the same are in writing, type-written copies of all these being preferred.

The Secretary should be made a standing member of the Committee on Publication in the future. The names of officers and Committees should be published as soon after the annual meeting as they are appointed and acceptances received, but we are of the opinion that when the proceedings of our Association are published annually, it is needless expense to publish the list of members separately.

The Committee do not claim the credit for the able manner in which the proceedings of the Chicago Convention are published in the splendid volume you all have received, but belongs to Dr. W. Horace Hoskins, of Philadelphia, Pa., who kindly consented to edit the volume, he being so thoroughly acquainted with all the matter published, and the Committee do sincerely thank him for his able work and for relieving them of a great task.

Respectfully submitted.

L. H. Howard, D.V.S.
A. T. Sellers, D.V.S.

